# Surveying Public Perceptions of Artificial Intelligence in Health Care in the United States: Systematic Review

**DOI:** 10.2196/40337

**Published:** 2023-04-04

**Authors:** Becca Beets, Todd P Newman, Emily L Howell, Luye Bao, Shiyu Yang

**Affiliations:** 1 Department of Life Sciences Communication University of Wisconsin–Madison Madison, WI United States; 2 Peking University HSBC Business School Shenzhen China

**Keywords:** artificial intelligence, AI, public opinion, public perception, health care

## Abstract

**Background:**

This paper reviews nationally representative public opinion surveys on artificial intelligence (AI) in the United States, with a focus on areas related to health care. The potential health applications of AI continue to gain attention owing to their promise as well as challenges. For AI to fulfill its potential, it must not only be adopted by physicians and health providers but also by patients and other members of the public.

**Objective:**

This study reviews the existing survey research on the United States’ public attitudes toward AI in health care and reveals the challenges and opportunities for more effective and inclusive engagement on the use of AI in health settings.

**Methods:**

We conducted a systematic review of public opinion surveys, reports, and peer-reviewed journal articles published on Web of Science, PubMed, and Roper iPoll between January 2010 and January 2022. We include studies that are nationally representative US public opinion surveys and include at least one or more questions about attitudes toward AI in health care contexts. Two members of the research team independently screened the included studies. The reviewers screened study titles, abstracts, and methods for Web of Science and PubMed search results. For the Roper iPoll search results, individual survey items were assessed for relevance to the AI health focus, and survey details were screened to determine a nationally representative US sample. We reported the descriptive statistics available for the relevant survey questions. In addition, we performed secondary analyses on 4 data sets to further explore the findings on attitudes across different demographic groups.

**Results:**

This review includes 11 nationally representative surveys. The search identified 175 records, 39 of which were assessed for inclusion. Surveys include questions related to familiarity and experience with AI; applications, benefits, and risks of AI in health care settings; the use of AI in disease diagnosis, treatment, and robotic caregiving; and related issues of data privacy and surveillance. Although most Americans have heard of AI, they are less aware of its specific health applications. Americans anticipate that medicine is likely to benefit from advances in AI; however, the anticipated benefits vary depending on the type of application. Specific application goals, such as disease prediction, diagnosis, and treatment, matter for the attitudes toward AI in health care among Americans. Most Americans reported wanting control over their personal health data. The willingness to share personal health information largely depends on the institutional actor collecting the data and the intended use.

**Conclusions:**

Americans in general report seeing health care as an area in which AI applications could be particularly beneficial. However, they have substantial levels of concern regarding specific applications, especially those in which AI is involved in decision-making and regarding the privacy of health information.

## Introduction

The potential applications of artificial intelligence (AI) in health care continue to gain attention because of their potential as well as challenges [1]. The potential uses of AI in health care contexts include clinical applications such as disease prediction and diagnosis [2-4] or the use of robots and robotic AI systems in caregiving [5], as well as broader public health applications such as disease outbreak prediction [6] or phone-based location tracking to combat the spread of diseases such as COVID-19 [7,8]. However, developers, health care providers, and policy makers are just starting to contemplate ethical concerns related to AI applications [9], and at a slower pace than the technology is being developed or deployed [10]. Policy makers often acknowledge the importance of engaging citizens in discourse and decision-making on AI but have not yet effectively moved to doing so.

For AI to fulfill its potential within health care, it must not only be adopted by physicians and health providers, but also by patients and other members of the public. To that extent, understanding public acceptance of technology is critical. This includes attitudes and perceptions related to the collection and use of health-related data, without which AI applications will not function [11]. Moreover, although there is expert consensus about what is and is not AI in the health care context [11], public views may be more mixed. Effective engagement and adoption of AI technologies in health care requires knowledge about what nonexperts think about this context. A more systematic understanding of public attitudes toward AI in health care will not only lead to better policy guidance for this topic but it can also inform more effective and responsible research and development [12].

Theories of technology adoption provide a framework for examining the various social, psychological, and behavioral variables that lead to the acceptance of new technologies. Specifically, the Technology Acceptance Model suggests that perceived usefulness and perceived ease of use influence one’s attitude toward the technology and intention to use the technology [13]. The importance of acknowledging different public views is critical given that the questions developers, health care providers, and policy makers think are most important to the public may be misaligned with empirical realities. Without a systematic understanding of the perceptions of usefulness, ease of use, and risks and benefits that AI technology in health care poses, the health care community runs the risk of providing the correct answers to the wrong questions. However, there is currently a lack of attention to public opinion of AI in health care and related issues, such as data privacy.

To address this need to better understand different public attitudes toward AI, we summarize key findings from existing nationally representative public opinion surveys on AI in the United States, with a focus on areas related to health care. Previous studies have explored specific stakeholders’ attitudes toward AI, including patients [14,15], medical practitioners [16,17], and political officials [18], among others. Our study is limited to nationally representative surveys to ensure the generalizability of the survey samples to the broader US population. We build on research examining public attitudes toward the application of AI in clinical settings [19,20] and expand the scope to AI and health care more broadly. We summarized the results of surveys focused on the use of AI in health care settings, examining public opinion on issues concerning the risks and benefits of AI, the use of AI in disease diagnosis and treatment, and related issues of data privacy and surveillance (eg, contact tracing). We also conducted and reported the results of secondary analyses on the associated data sets, when available, to understand differences across key demographics. Our primary research question is as follows: what does existing survey research examining the US publics’ attitudes toward AI applications in health care reveal about the challenges and opportunities for more effective and inclusive engagement on the use of AI in health settings?

## Methods

### Study Design

We conducted a systematic review of public opinion surveys, reports, and peer-reviewed journal articles on public perceptions of AI in the United States. Our findings were reported following the PRISMA (Preferred Reporting Items for Systematic Reviews and Meta-Analyses) guidelines [21].

### Eligibility Criteria

We included studies that (1) are nationally representative US public opinion surveys and (2) include at least one or more questions about attitudes toward AI in health care contexts in the United States. Our criteria for nationally representative surveys included surveys that use either probability or nonprobability sampling, which have been weighted on key demographic measures (eg, age, gender, and education) to match the demographics of the US population [22].

We excluded studies that did not use representative survey designs, such as studies that reported on convenience samples or were not clearly identified as representative samples [23-25], as well as those that exclusively focused on a specific subpopulation. In addition, we excluded studies that were based on populations outside of the United States, which did not include questions related to the specific AI health focus of this review, or those that were not published in English.

### Search Strategy

The studies included in this review were identified through searches on Web of Science, PubMed, and Roper iPoll. The searches used a combination of terms related to AI, public opinion, and health. The full search strings used for PubMed, Web of Science, and Roper iPoll are included in Multimedia Appendix 1. The searches were limited to studies published in English between January 1, 2010, and January 31, 2022. The final search of all 3 databases was conducted on February 6, 2022.

### Study Selection and Data Collection

A total of 2 members of the research team independently screened the studies for inclusion, based on the selection criteria. For the Web of Science and PubMed search results, we screened the study titles, abstracts, and methods to determine their inclusion. On the basis of our eligibility criteria, studies with a nationally representative US survey sample were included, whereas all others were excluded (eg, non–US samples or studies not clearly identified as representative). For the Roper iPoll search results, individual survey items were assessed for relevance to the AI health focus, and the survey details were screened to determine whether they used a nationally representative US sample.

For each study, we collected data on the survey title, the month and year the survey was fielded, the sampling method, the sample size, information about how the survey sample was weighted, details on who conducted the survey, and the general topical focus of the survey. We also recorded the relevant survey questions relating to the applications of AI in health care and the response data for each question.

### Data Reporting

For each survey included in this review, we reported the available descriptive response statistics for the relevant AI health-related questions (eg, the percentage of respondents who agreed to a particular statement). These data did not include any effect measures. Because this study was limited to public opinion surveys, typical methods to assess the risk of bias in systematic reviews of clinical data do not apply to our descriptive cross-sectional data [26].

### Secondary Data Analyses

Given the growing concerns about equitable distribution of the risks and benefits of AI applications, we also performed secondary analyses on publicly available data sets to provide findings on attitudes across different demographic groups.

Specifically, we examined response patterns broken down by demographic variables such as race, gender, age, education, and income, whenever such demographic variables were available in the data sets. Among the 11 nationally representative surveys we reviewed, only 3 made their full data sets publicly available for secondary analysis, including 2 data sets from the Pew Research Center [27,28] and 1 from Monmouth University [29]. In addition, we drew on a recent data set fielded by the authors’ research group, Science Media and the Public (SCIMEP) [30].

For each of the available data sets, we downloaded publicly available data and applied the weights provided for each sample before running the descriptive analyses of the demographic subgroups. Because survey structure and question wording are unique to each data set, survey question analysis was limited to individual questions and not synthesized across studies. Data analysis was conducted using IBM SPSS Statistics (IBM Corp), and data were visualized using Microsoft Excel.

## Results

### Overview

**Figure 1:**
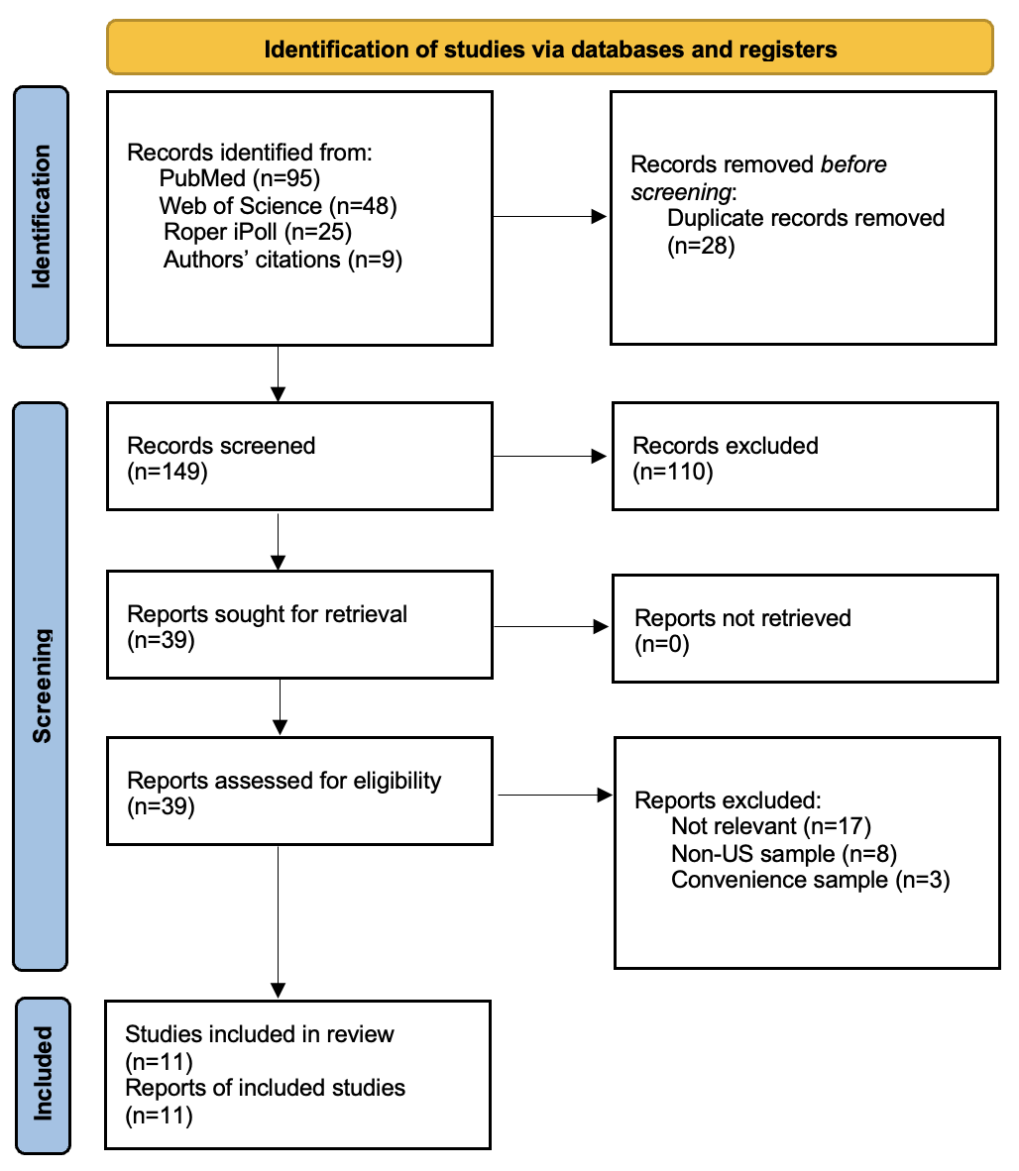
PRISMA (Preferred Reporting Items for Systematic Reviews and Meta-Analyses) flow diagram of records identified and included in the study.

We identified 175 records, of which 39 were assessed for inclusion based on our selection criteria (Figure 1). Our final sample included 11 nationally representative surveys (Table 1). The surveys focus on the American publics' general knowledge, experience, and views of AI in health care. They included questions that tap into issues related to general knowledge and attitudes toward AI, AI-based treatment and diagnosis, and data sharing and privacy.

The results are reported thematically, based on the questions included in the surveys. For each question, we report the wording of the question, total survey sample size, and response percentages corresponding to each question. The descriptive statistics reported throughout correspond to the individual surveys. For the 4 surveys where we had access to the data sets, we provided additional demographic breakdowns of the survey responses (eg, by race or education level) in the relevant sections. We summarize our key findings as follows.

**Table 1:** Overview of nationally representative surveys of US public opinion of artificial intelligence and health care.

Survey	Year	Sampling method	Weighted on	Topic
Pew Research Center^a^ [31]	February 2014	Nonprobability sample^b^; telephone interview, cell phone and landline. Conducted by Princeton Survey Research Associates International (n=1001)	Age, education, gender, race, Hispanic origin, population density, region (US Census definitions), telephone use	Future; health; medicine; science
Monmouth University Polling Institute^a^ [29]	March-April 2015	Nonprobability sample; telephone interview, cell phone and landline. Conducted by Monmouth University Polling Institute (n=1005)	Age, education, gender, race, region (US Census)	Information; science
Vanity Fair/60 Minutes [32]	March 2016	Nonprobability sample; telephone interview, cell phone and landline. Conducted by SSRS on behalf of CBS News (n=1021)	US Census figures on demographic variables (unspecified)	Communication technology; mood; religion; technology
Ghafur et al [33]	2018	Nonprobability sample, from a web-based panel; web-based survey. Conducted by YouGov (n=1114)	Age, education, gender, race	Public perceptions on data sharing
Zhang and Dafoe [34]	June 2018	Nonprobability sample, from a web-based panel; web-based survey. Conducted by YouGov (n=2000)	Age, education, gender, race	American public’s attitudes toward AI^c^ and AI governance
Pew Research Center^a^ [27]	June 2019	Nonprobability sample, from probability-based panel; web-based survey. Conducted by Ipsos (n=4272)	Age, education, gender, race, Hispanic origin, Hispanic nativity, home internet access	Privacy and surveillance
Rock Health and Stanford Center for Digital Health [35]	July-August 2019	Nonprobability sample, from member panel; web-based survey. Conducted by Toluna (n=4000)	Age, gender, geographic region, income, race	Annual digital health consumer adoption
SCIMEP^a,d^ [30]	2020	Nonprobability sample, from a web-based panel; web-based survey. Conducted by YouGov (n=2700)	Age, education, gender, race	Public attitudes toward AI
Pew Research Center^a^ [28]	April 2020	Nonprobability sample, from probability-based panel; web-based survey. Conducted by Ipsos (n=4917)	Age, education, gender, race, Hispanic origin, Hispanic nativity, home internet access	Responses to the new coronavirus outbreak
Rock Health and Stanford Center for Digital Health [36]	September-October 2020	Nonprobability sample, from member panel; web-based survey. Conducted by Toluna (n=7980)	Age, gender, geographic region, income, race	Annual digital health consumer adoption
Pew Research Center [37]	November 2021	Nonprobability sample, from probability-based panel; web-based survey. Conducted by Ipsos (n=10,260)	Age, education, gender, race, Hispanic origin, Hispanic and Asian nativity, years lived in the United States	Internet and science

^a^Indicates the survey data used for secondary analyses.

^b^Nonprobability sample: poststratification weights for each sample were applied to the analyses to improve representativeness.

^c^AI: artificial intelligence.

^d^SCIMEP: Science Media and the Public.

### General Knowledge and Attitudes Toward AI in Health Care

#### Key Findings

Although most Americans have heard of AI, they are less aware of the specific health applications of AI. Americans anticipate that medicine is most likely to benefit from advances in AI compared with other fields such as military science. The anticipated benefits vary depending on the type of application; however, they are less clear for applications that involve AI making health decisions for people.

#### Knowledge, Awareness, and Experience With AI in General

A total of 2 surveys included questions related to familiarity and experience with AI generally. Most Americans have at least some familiarity with AI. A 2015 survey by the Monmouth University Polling Institute (n=1005) found that 70% of Americans had heard of the term “artificial intelligence” or “AI” [29]. For most, direct experience with AI comes through their cell phones and smart personal assistants, such as Alexa (Amazon) and Cortana (Microsoft Corporation), Siri (Apple), and Google Assistant (Google). A 2020 survey by the SCIMEP research group (n=2700) found that 83% of Americans used smartphones and 29% of Americans reported using smart personal assistants “at least once” to “a few times a week” [30].

However, poll data suggest that there is still ample room for Americans to learn more about AI. In the same 2015 Monmouth University survey (n=1005), only 12% of Americans reported having read or heard “a lot” about recent advancements in AI technology, whereas 88% of the Americans had read or heard “a little” or “nothing at all” [29]. As with many issues, people may report opinions on AI based on the information they hold at these early stages as public familiarity with AI increases.

#### Perceived Applications, Benefits, and Risks of AI in Health Care

A total of 3 surveys included questions related to the applications, risks, and benefits of AI in health care. Health care is a prominent area in which people anticipate the benefits of AI development and application. In a 2016 Vanity Fair/60 Minutes survey (n=1021), respondents were asked which fields were most likely to benefit from advances in AI [32]. Slightly fewer than half (44%) of the respondents answered “medicine,” topping the list. For comparison, 23% answered “military science” and 13% answered “automobile manufacturing” [32].

Despite the view that health care as a field will likely benefit from AI advancement, Americans are less aware of the specific health applications of AI (Figure S1 in Multimedia Appendix 2). The 2020 SCIMEP survey (n=2700) found that about half of Americans (48%) had either “little” or “no” awareness of using AI to diagnose diseases more efficiently, and only one-fifth of Americans (19%) were “quite a bit” or “a great deal” aware of use of AI in disease diagnosis [30].

When it comes to the likelihood of AI applications to improve individuals’ health, the 2020 SCIMEP survey (n=2700) found that Americans’ views were more divided, with 34% responding that AI is “likely,” “very likely,” or “certainly” going to improve health, 35% responding “somewhat likely,” and 31% responding “unlikely,” “very unlikely,” or “not at all likely” [30]. Consistent with trends of general support for AI [34], the SCIMEP survey found that the perceived likelihood of AI improving individuals’ health was positively associated with their education and income [30]. Among Americans with at least a 4-year college degree, for example, 43% said that AI will “likely” to “certainly” improve health. That is twice the number of respondents who said it is “unlikely” to “not at all likely” (21%) in that same group. In comparison, only 31% of those who had a high school diploma or less said that AI will “likely” to “certainly” improve health. Likewise, as household income increased, the perceived likelihood of AI improving individuals’ health also increased. Among Americans whose annual household income was over US $100,000, twice as many people were “likely” to “certainly” (45%) to believe that AI would improve health compared with “unlikely” to “not at all likely” (21%). Of those with a household income of US $30,000 to US $70,000, only 33% responded “likely” to “certainly” [30].

Although Americans generally have positive views about using AI technology to advance public health and medical fields, their attitudes become more cautious when it comes to allowing AI to make important personal health decisions. A 2021 survey by Pew Research Center (n=10,260) asked how “excited or concerned” respondents would be “if artificial intelligence computer programs could diagnose medical problems” [37]. Results were divided, with 40% of respondents either “very” or “somewhat” excited, 24% equally concerned and excited, and 35% either “somewhat” or “very” concerned. In another example, the 2016 Vanity Fair/60 Minutes survey (n=1021) found that only 8% of respondents felt comfortable letting a computer with AI decide on their end-of-life care [32]. Given the range of potential health applications of AI, additional research is required to explore how specific perceived risks and benefits vary across uses and contexts.

### AI-Based Treatment and Diagnosis

#### Key Findings

Specific application goals, such as disease prediction, diagnosis, and treatment, matter to Americans’ attitudes toward AI in health care. Secondary analyses showed clear demographic differences regarding acceptance of some of these applications, such as the use of AI for mental health monitoring and treatment. Americans also recognize the importance of future governance challenges related to the use of AI for disease diagnosis.

#### Disease Prediction and Diagnosis

A total of 3 surveys included questions on disease prediction and diagnosis. The 2020 SCIMEP survey (n=2700) data indicated a salient difference between Black and White Americans in their concern about AI worsening discrimination against people based on health risks. Among Black Americans, 40% were “concerned” or “very concerned” about AI worsening discrimination on the basis of individuals’ health risks, compared with 33% of Black Americans who were “slightly” or “not at all” concerned [30]. In contrast, 30% of White Americans were “concerned” or “very concerned” about AI worsening discrimination against people based on health risks, whereas 44% of White Americans were “slightly” or “not at all” concerned about such worsening discrimination.

Surveys that focus on the use of AI for mental health monitoring and treatment, however, find public opinion to be divided somewhat differently among Black and White Americans, with White Americans being more concerned about such uses (Figure S2 in Multimedia Appendix 2). The 2019 Pew Research Center poll (n=4272) asked whether social media companies should monitor their users’ posts for signs of depression to identify people who are at risk of self-harm and connect them to counsel [27]. Overall, 45% of Americans found this use of personal data to be unacceptable compared with 27% who found it acceptable. Among those, half of the White Americans (50%) found it unacceptable, whereas only a quarter (24%) found it acceptable. In contrast, Black Americans showed slightly higher acceptance (41%) than unacceptance (34%) [27].

The 2020 SCIMEP survey (n=2700) asked a similar question regarding companies using AI and personal information to make mental health predictions, although in this case, insurance providers use AI to predict whether a person will develop depression in the future [30]. Consistent with the 2019 Pew Research Center data, White Americans showed higher concern (51% “concerned” or “very concerned”) about this use than Black Americans (43% “concerned” or “very concerned”) and Hispanic Americans (39% “concerned” or “very concerned”). Education levels mattered as well across all races. More than half of Americans with a 4-year college degree or higher education (55%) were “concerned” or “very concerned” about the application of predicting depression by insurance companies, without substantial differences across racial groups [30].

Americans express a desire for transparency in the management of AI disease-diagnosis technology. The 2019 Zhang and Dafoe survey (n=2000) found that nearly half of Americans (44%) agreed that accuracy and transparency in AI used for disease diagnosis would likely be an AI governance challenge that will impact large numbers of people in the United States in the next 10 years [34]. Among those, 10% thought this governance challenge would be “very likely,” 13% “likely,” and 21% “somewhat likely.” More than three-quarters of Americans also viewed AI applications in disease diagnosis as very important (56%) or somewhat important (22%) for technology companies and governments to manage carefully [34]. The perceived importance of carefully managing the challenge of AI disease diagnosis is comparable with the perceived importance of managing challenges in AI applications in surveillance (59% “very important” and 19% “important”) and a bit lower than that of data privacy (64% “very important” and 18% “important”).

#### Robotic Caregivers and AI-Assisted Health Care Decision-Making

In addition to health monitoring and diagnosis tools supported by AI, 3 surveys included questions about AI providing treatment in the literal sense of AI robots as caregivers (Figure S3 in Multimedia Appendix 2). Americans seem to be skeptical of the value of robots and AI in caregiving and care-related decision-making, at least as replacements for human caregivers. A 2014 Pew Research Center survey (n=1001) found that the majority (65%) of respondents thought it would be a change for the worse, if lifelike robots became the primary caregivers for the older adults and people in poor health, whereas 28% of respondents thought it would be a change for the better [31]. There were no substantial gaps in attitudes toward robotic caregivers across racial and gender groups.

Americans hold similarly unfavorable views regarding the use of AI robots in hospital-level decision-making and resource allocation. The 2018 Ghafur et al [33] survey (n=1114) found that only 38% of respondents were happy to receive AI-assisted health advice. In the 2015 Monmouth University survey (n=1005), 65% of Americans said it was a “bad idea” to use robotic nurses with AI to diagnose situations and decide when to administer medicine for bed-ridden patients, whereas only about 3 in 10 thought it was a “good idea” [29]. Broken down by demographics, Hispanic (49%) and Asian Americans (44%) had the most favorable views of this application of AI, followed by Black (33%) and White Americans (27%). A gender gap exists here as well, with 38% of men thought it was a good idea, whereas only 24% of women agreed [29].

### Data Sharing and Privacy

#### Key Findings

Data sharing and privacy associated with health-related data are key concerns among Americans. Most Americans reported wanting control over their personal health data. Willingness to share their personal health information largely depends on which organization or institutional actor collects the data, the intended purpose of data sharing, and the specific groups being tracked. Americans report lower trust in commercially oriented organizations, such as technology companies, compared with doctors or pharmacists. Americans also express a desire to control their health information, especially concerning how others access and share it. Demographics such as race and education shape attitudes regarding data sharing and privacy.

#### Control of Personal Information

A total of 3 surveys included questions on control over personal health data. In a 2019 survey by the Rock Health and Stanford Center for Digital Health (n=4000) on consumer adoption of digital health technologies, most respondents (82%) expressed a desire to control those who had access to their health data. Similarly, 81% said they “agree” or “strongly agree” that “I should be told what data had been collected about me” [35].

Americans also want control over the life span of their personal health data (Figure S4 in Multimedia Appendix 2). In a 2019 Pew Research Center survey (n=4272), 71% of respondents agreed that all Americans should have the right to have their personal medical data collected by a health provider permanently deleted [27]. White Americans (74%) were more likely to agree that they should have the right to remove personal medical data compared with Black (60%) and Hispanic (66%) Americans. There was a prominent education-based divide within the Black American community. Black Americans who had obtained at least a college degree were more likely to agree that they should have the right to delete their medical data (79%) than those who only had a high school diploma or less (46%) [27]. In this survey, education did not similarly predict attitudes for White Americans.

Moreover, the intended purpose of health data sharing is also a key influence on attitudes toward sharing health data. In the same 2019 Pew Research Center survey (n=4272), respondents were asked whether they thought “DNA testing companies sharing their customers’ genetic data with law enforcement agencies in order to help solve crimes” was an acceptable or unacceptable use of their data [27]. About half of the White (51%) and Hispanic (50%) Americans thought it was acceptable. Black Americans (46%) showed slightly less acceptance compared with Americans of other races. In addition, Black Americans with a college degree showed lower acceptance (38%) than their Black counterparts who had attended college (45%) or had a high school diploma or lower (50%) [27]. The 2020 SCIMEP survey (n=2700) similarly found that Black Americans had greater concern (46% “concerned” or “very concerned”) about DNA testing companies sharing their customers’ genetic data with law enforcement agencies to help solve crimes than White (40% “concerned” or “very concerned”) or Hispanic Americans (31% “concerned” or “very concerned”) [30].

In contrast, the 2019 Pew Research Center survey (n=4272) found that when the purpose of data sharing was “makers of a fitness tracking app sharing their users’ data with medical researchers seeking to better understand the link between exercise and heart disease,” White Americans had lower acceptance (40%) than their Black and Hispanic counterparts (47% and 49%, respectively) [27]. Across all racial groups, Hispanic Americans with higher levels of education (some college, 58%; college degree or more, 60%) or an income of US $30,000 to US $74,999 (64%) had the highest levels of acceptance.

#### Trust in Who Controls Personal Information

Three surveys included questions related to trust in controlling personal health data. Surveys indicate that the willingness to share personal health data largely depends on who the data-receiving body is (Figure S5 in Multimedia Appendix 2). A 2018 survey by Ghafur et al [33] (n=1114) indicated that Americans were most willing to share their anonymized health information with personal doctors (61%), followed by one’s family (41%), academic or medical research institutions (26%), and pharmacists (25%). They were least willing to share with technology companies, such as Google, Amazon (4%), or any other commercial companies (3%). In addition, 15% of respondents were unwilling to share their anonymized health information with anyone [33].

A 2020 survey by Rock Health and the Stanford Center for Digital Health (n=7980) found that consumers were most willing to share their health information with their physicians (72%), followed by their families (52%), health insurers (53%), pharmacies (46%), research institutions (35%), health technology companies (25%), pharmaceutical companies (22%), their employers (15%), government organizations (12%), and technology companies (11%) [36]. The same question was asked in a 2019 survey by Rock Health (n=4000) and the findings were consistent [35].

The 2020 Rock Health survey (n=7980), which was conducted during the COVID-19 pandemic, also asked respondents about their willingness to share their COVID-19 results with these groups [36]. One-third (32%) of the respondents were willing to share their COVID-19 results with their employers, and approximately one-fourth (23%) were willing to share them with government organizations. This is twice the number of respondents willing to share their general, broader health information with these entities (15% and 12%, respectively) [36].

However, overall, these findings suggest that respondents’ willingness to share personal health information decreases as the data-receiving institution becomes more commercially oriented.

#### Location Tracking and Privacy

One survey included questions related to the location tracking of health-related data. The results of this survey suggest that views on whether such tracking is useful or acceptable are mixed. A survey by the Pew Research Center conducted in April 2020 (n=4917) asked respondents, “if the government tracked people’s locations through their cell phones during the coronavirus outbreak, do you think this would help a lot, a little, or not make a much difference in limiting the spread of the virus?” [28]. Most US adults (60%) said that such actions would not make much of a difference in limiting the spread of the virus.

Regarding acceptability, people have nuanced views depending on who is being tracked and for what purpose. In the scenario of governments using people’s cell phones to track their location, Americans were relatively tolerant of the use for those who have “tested positive for the coronavirus in order to understand how the virus may be spreading,” with just over half (52%) of respondents reporting it was either “very” or “somewhat” acceptable (24% and 28%, respectively) [28]. This decreased slightly for those who may have “had contact with someone who tested positive for the coronavirus,” with 45% responding that it was “very” or “somewhat” acceptable (19% and 26%, respectively). The lowest acceptance was for location data use to “ensure [people] are complying with experts’ advice on limiting social contact during the coronavirus outbreak,” with 37% responding “very” or “somewhat” acceptable (14% and 23%, respectively) [28].

White Americans had the lowest acceptance for all 3 location-tracking strategies among racial groups. Nearly half of White Americans (47%) reported it was either “very” or “somewhat” acceptable (19% and 28%, respectively) for the government to track people who tested positive for coronavirus [28]. In comparison, 56% of Black Americans said that it was “very” or “somewhat” acceptable (34% and 22%, respectively) and 66% of Hispanic Americans said it was “very” or “somewhat” acceptable (33% and 32%, respectively). Similarly, only about a third (31%) of White Americans thought the use of location tracking was “very” or “somewhat” acceptable (12% and 19%, respectively) to ensure limiting social contact. This was much lower than the 45% of Black Americans who said it was “very” or “somewhat” acceptable (24% and 21%, respectively) and 55% of Hispanic Americans who said it was “very” or “somewhat” acceptable (23% and 32%, respectively) [28].

These distinctions raise interesting and important questions for communication and engagement on how differences in experience with the pandemic, governments, technologies, and other factors vary depending on one’s race to shape views of AI use and acceptability.

## Discussion

### Overview

The aim of this study was to review existing survey research examining the US publics’ attitudes on AI applications in health care, and to reveal the potential challenges and opportunities for broad acceptance of the technology in the health care context. We found that a small number of nationally representative surveys (n=11) capture public perceptions of the different aspects, applications, and potential impacts of AI in public health settings. Although most Americans have heard of AI, they are less aware of specific health applications. Americans anticipate that medicine is likely to benefit from advances in AI, however, the anticipated benefits vary depending on the type of application. Specific application goals, such as disease prediction, diagnosis, and treatment, matter to Americans’ attitudes toward AI in health care. Finally, the related issue of data sharing and health data privacy is a key concern, with most Americans wanting control over their personal health data.

Americans, in general, perceive health care as an area in which AI applications could be beneficial for the field. However, there are substantial concerns regarding specific applications and their benefits to individuals. This is particularly evident for applications in which AI is involved in decision-making and regarding the privacy and use of health information. Data on the perceived usefulness of AI applications in health care are important for understanding current and future patient attitudes toward and intentions to use the technology [13]. Despite low overall levels of familiarity with AI as a concept or through specific applications, many Americans, especially those with higher education, were aware of and concerned about personal lack of control over how their data are collected and used and by whom. The majority were also hesitant to agree that AI with primary decision-making power in health care contexts would be a good idea. Assuaging public concern regarding health data privacy and the role of personal data in AI technologies is an important dialogue to cultivate between health care providers, developers, policy makers, and current and future patients.

Our secondary analyses of the available public opinion data consistently found that levels of concern and acceptability vary across demographic groups but not always in the same direction. Across racial demographics, White Americans were typically more likely to believe that people should have the right to delete their personal health data and were more likely to be concerned about sharing data with researchers, using location tracing for combating the spread of COVID-19, or insurance or social media companies using AI and personal data for predictive purposes. Black Americans, however, were more likely than other groups to be concerned about law enforcement having access to personal data. Polls indicate that most Americans, regardless of their gender or race, have very little trust in companies when it comes to using personal health data.

Americans with higher educational attainment reported higher levels of acceptance of sharing data with medical researchers and lower levels of acceptance of sharing data with commercial companies and law enforcement. They are more likely to report that AI technologies improve individuals’ health and that people should have the right to their personal health data. This is notable because much of the health data in the United States are in the hands of private companies [33].

Although questions about the responsibility and rights involved in data collection and use are becoming increasingly important, these are reflected in only a few existing public opinion surveys. While surveys of experts in technology development and policy found that experts frequently mentioned potential inequalities caused by AI in health care [38], surveys have rarely tapped into different publics' views on inequity issues concerning AI. Furthermore, although trust in different institutions and social groups is likely an important component of perceptions and use of AI in health contexts, little of the survey research that we found included items measuring Americans’ trust levels in institutions and social groups involved in AI development itself—a stage in the life span of AI applications that has implications for the subsequent use of AI and related data collection, and one in which early feedback and engagement with public is especially vital.

### Limitations

Before discussing the implications of our findings, it is important to acknowledge the limitations of this study. The first limitation relates to the claim of representativeness in the included surveys. We limited our search to nationally representative surveys, meaning those based on probability or nonprobability sampling. Each of the 11 surveys included in our review uses a nonprobability sample weighted on various demographic variables. Weighting this way will improve sampling estimates; however, certain populations such as those less likely to participate in web-based panels may still be underrepresented, biasing the results [39]. Second, this study was limited to surveys of the US population. Limiting to the United States was intentional, given the unique group-level and social dynamics that come into play in the US health care system. For example, the cultural and demographic differences that impact medical mistrust in the United States [40] likely contribute to attitudes toward different AI applications, data sharing, and privacy. These factors make it challenging to draw comparisons with other populations. Third, the definitions of AI used in each of the surveys varied (see Multimedia Appendix 3 for a list of definitions). Fourth, it is possible that we missed surveys that were not included in the PubMed or Web of Science databases. Finally, this study’s focus on public opinion data differs from clinical data often included in systematic reviews related to health topics. Although we sought to align our data within the PRISMA framework [21], there are some typical reporting factors that are not included (eg, effect sizes).

Despite these limitations, this study offers an important look at what is currently known and unknown about Americans’ attitudes toward AI applications in health care. Future research on public perceptions of AI should work to understand different public views regarding how, why, and for and by whom personal health data should be collected and used. Going forward, more work is needed to understand different publics' awareness and opinion across the stages of the AI system life cycle, which includes the following: (1) design, data collection, and model building; (2) verification and validation; (3) deployment; and (4) operation and monitoring of AI systems. As AI applications become more widespread in health care contexts, it is critical to understand public perceptions and how public attitudes align with the issues that developers, health care providers, and policy makers consider most important. Moreover, understanding public concerns is a necessary step toward the responsible development of AI.

## Appendix

Multimedia Appendix 1 Search string for PubMed, Web of Science, and Roper iPoll.

Multimedia Appendix 2 Comparing survey results.

Multimedia Appendix 3 Definitions of artificial intelligence used in included surveys.

## Abbreviations

AI: artificial intelligence
PRISMA: Preferred Reporting Items for Systematic Reviews and Meta-Analyses
SCIMEP: Science Media and the Public
