# Supplementary material for: Surveying Public Perceptions of Artificial Intelligence in Health Care in the United States: Systematic Review
Source: J Med Internet Res. 2023 Apr 4;25:e40337. doi: 10.2196/40337 (PMC10131909; doi:10.2196/40337)
Supplement: Multimedia Appendix 1 [file jmir_v25i1e40337_app1.pdf]

Multimedia Appendix 1
Search String for PubMed, Web of Science, and Roper iPoll

PubMed

(“artificial intelligence” OR “AI” OR “machine learning”) AND (“public opinion” OR “public attitudes” OR “public views” OR "public perceptions") AND (health*).

Web of Science

(TS=(("artificial intelligence" OR "AI" OR "machine learning") AND ("public opinion" OR "public attitudes" OR "public views" OR "public perceptions") AND (health*)))

Roper iPoll

(“artificial intelligence” OR “AI” OR “machine learning” OR robot*)
