# Supplementary material for: Surveying Public Perceptions of Artificial Intelligence in Health Care in the United States: Systematic Review
Source: J Med Internet Res. 2023 Apr 4;25:e40337. doi: 10.2196/40337 (PMC10131909; doi:10.2196/40337)
Supplement: Multimedia Appendix 2 [file jmir_v25i1e40337_app2.pdf]

Multimedia Appendix 3
Results Figures

Contents

Figure S1. Awareness and perceptions of AI in healthcare …………………….……………… 2

Figure S2. Attitudes toward the use of AI to predict disease diagnosis ……………………….… 3

Figure S3. Attitudes toward robotic caregivers …….…………………………………….. 4

Figure S4. Perceptions of AI and the control of personal information ………………………... 5

Figure S5. Trust in who controls personal information …………...………………………..…. 6

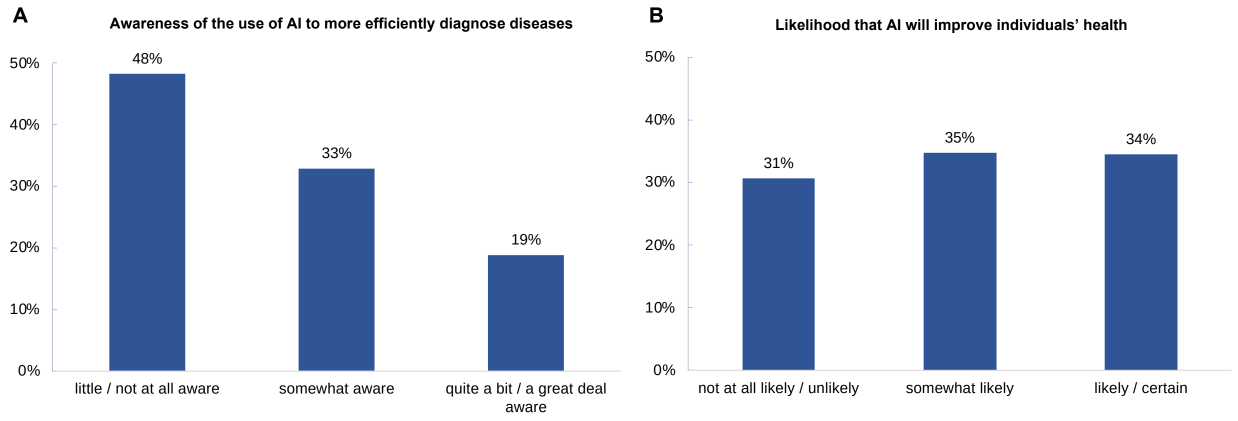

Figure S1. Awareness and perceptions of AI in health care. (A) Question wording: "How aware are you of the following developments involving AI, if at all… using AI to more efficiently diagnose diseases" (2020 SCIMEP survey, n=2700) [30]. (B) Question wording: "How likely do you think AI will... improve individuals' health" (2020 SCIMEP survey, n=2700) [30].

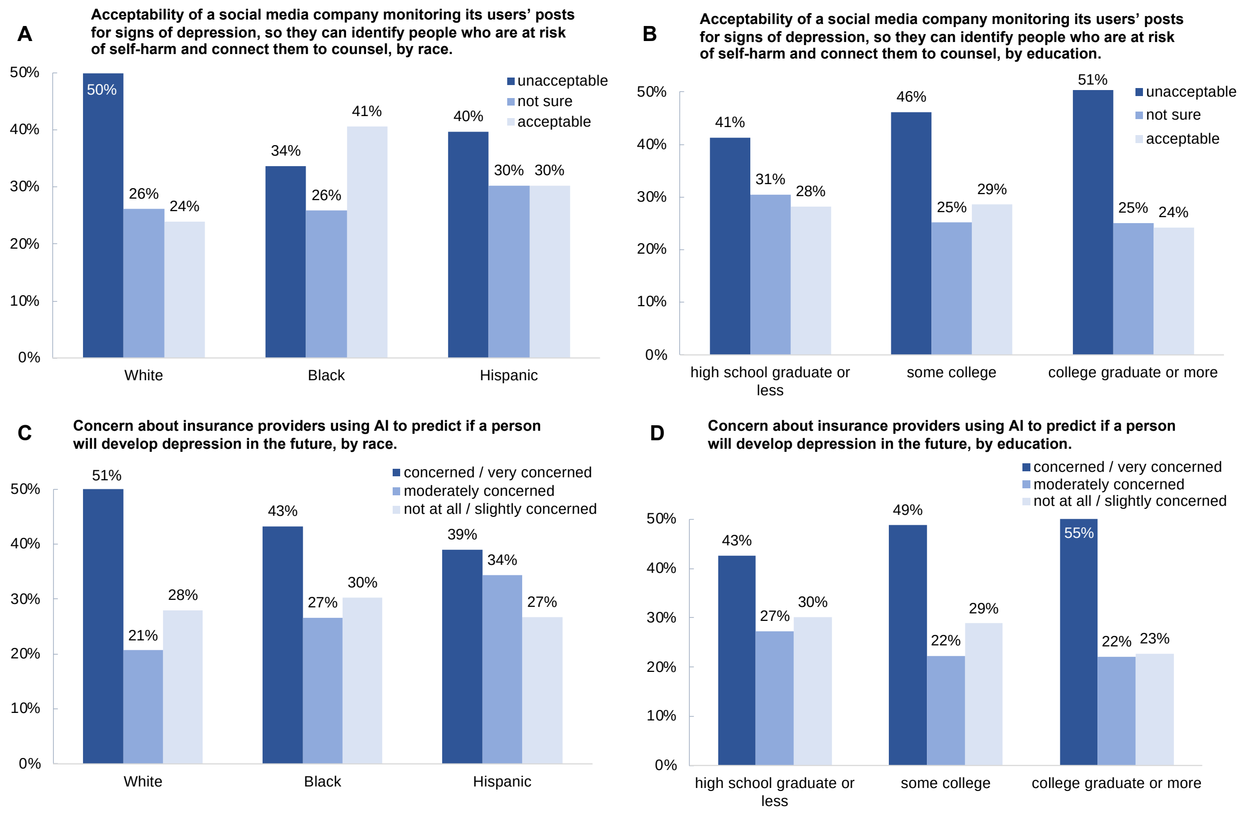

Figure S2. Attitudes toward the use of AI to predict disease diagnosis. (A-B) Question wording: "Do you think the following uses of data/information are acceptable or unacceptable? Social media company monitoring its users' posts for signs of depression, so they can identify people who are at risk of self-harm and connect them to counsel" (2019 Pew Research Center, n=4272) [27]. (C-D) Question wording: "How concerned are you about the following potential uses of AI? Insurance providers using AI to predict if a person will develop depression in the future" (2020 SCIMEP survey, n=2700) [30].

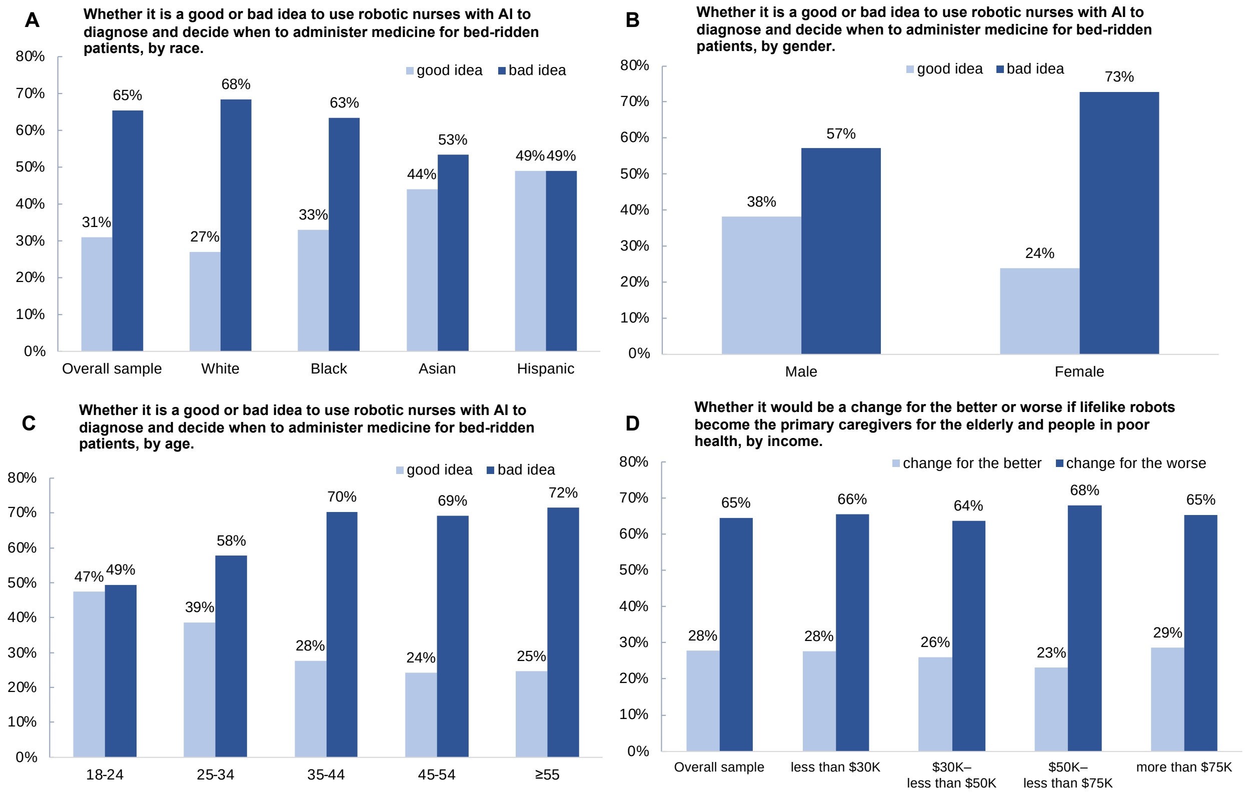

Figure S3. Attitudes toward robotic caregivers. (A-C) Question wording: “I am going to read you some examples of how artificial intelligence can be used. For each, please tell me whether you think it would be a good idea or bad idea to use machines with artificial intelligence to do these tasks. Robotic nurses with AI to diagnose and decide when to administer medicine for bed-ridden patients—Is that a good idea or bad idea?” (2015 Monmouth University, n=1005) [29]. (D) Question wording: “Next, here are some things that might happen in the next 50 years. For each, tell me if you think it would be a change for the better or a change for the worse if this happens. How about if lifelike robots become the primary caregivers for the elderly and people in poor health” (2014 Pew Research Center, n=1001) [31].

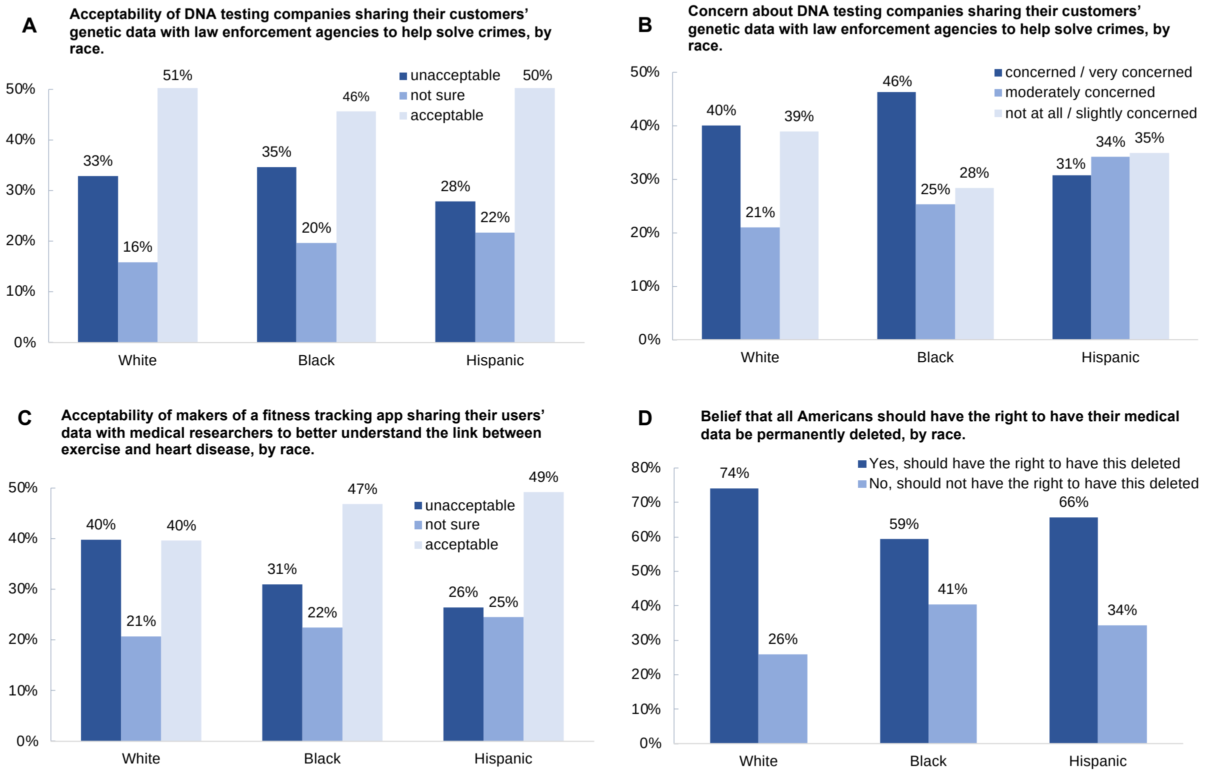

Figure S4. Perceptions of AI and the control of personal information. (A) Question wording: "Do you think the following uses of data/information are acceptable or unacceptable? DNA testing companies sharing their customers' genetic data with law enforcement agencies in order to help solve crimes" (2019 Pew Research Center, n=4272) [27]. (B) Question wording: "How concerned are you about the following potential uses of AI? DNA testing companies sharing their customers' genetic data with law enforcement agencies in order to help solve crimes" (2020 SCIMEP survey, n=2700) [30]. (C) Question wording: "Do you think the following uses of data/information are acceptable or unacceptable? Makers of a fitness tracking app sharing their users' data with medical researchers seeking to better understand the link between exercise and heart disease" (2019 Pew Research Center, n=4272) [27]. (D) Question wording: "Do you think that ALL Americans should have the right to have the following data about themselves be permanently deleted by the people or organizations who have that information? Medical data collected by a health provider" (2019 Pew Research Center, n=4272) [27].

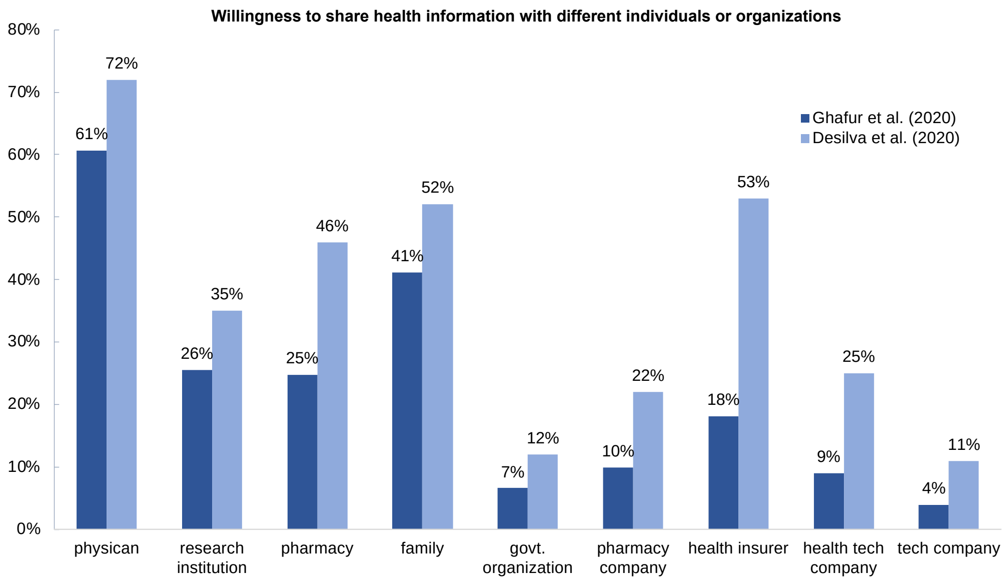

Figure S5. Trust in who controls personal information. Question wording: "With who, if anyone, would you be willing to share your anonymised personal health information" (Ghafur et al, n=1114) [33]. Question wording: "Please indicate which of the following individuals or organizations you would be willing to share your health information with (e.g., your medical records, test results, prescription drug history, genetic information, and physical activity data)" (2020 Rock Health, n=7980) [36].
