# Supplementary material for: Surveying Public Perceptions of Artificial Intelligence in Health Care in the United States: Systematic Review
Source: J Med Internet Res. 2023 Apr 4;25:e40337. doi: 10.2196/40337 (PMC10131909; doi:10.2196/40337)
Supplement: Multimedia Appendix 3 [file jmir_v25i1e40337_app3.pdf]

Multimedia Appendix 2
Definitions of AI Used in Surveys

Survey	**Year**	**AI Definition**
Pew Research Center* [31]	2014 February	No definition provided.
Monmouth University Poll* [29]	2015 March – April	“Artificial intelligence is the ability of computers and machines to carry out decision-making and thought processes similar to humans, sometimes referred to as computers being able to think for themselves.”
Vanity Fair/60 Minutes [32]	2016 March	“The term Artificial Intelligence refers to the ability of computers and machines to perform tasks that normally require human intelligence.”
Ghafur et al. [33]	2018	“by "artificial intelligence software", we mean the use of algorithms and software to aid the analysis of complex medical data with the aim of understanding the relationships between prevention or treatment techniques and patient outcomes.”
Zhang & Dafoe [34]	2018 June	“Artificial Intelligence (AI) refers to computer systems that perform tasks or make decisions that usually require human intelligence. AI can perform these tasks or make these decisions without explicit human instructions.”
Pew Research Center* [27]	2019 June	No definition provided.
Rock Health and Stanford Center for Digital Health [35]	2019 July – August	No definition provided.
SCIMEP* [30]	2020	“Artificial intelligence (AI) typically refers to the ability of computers and machines to perform tasks that normally require human intelligence. AI can perform these tasks or make these decisions without explicit human instructions"
Pew Research Center* [28]	2020 April	No definition provided.
Rock Health & Stanford Center for Digital Health [36]	2020 September – October	No definition provided.
Pew Research Center [37]	2021 November	“Artificial intelligence computer programs are designed to learn tasks that humans typically do, for instance recognizing speech or pictures.”
